# Fibrodysplasia Ossificans Progressiva: A Man Turned to Stone

**DOI:** 10.7759/cureus.61661

**Published:** 2024-06-04

**Authors:** Nadina Kurtanović, Ena Gogić, Alen Džubur, Edin Begić, Asja Bijedić

**Affiliations:** 1 Physical Medicine and Rehabilitation, Health Institution Spa Gata Bihać, Bihać, BIH; 2 Physical Medicine and Rehabilitation, Clinical Center University of Sarajevo, Sarajevo, BIH; 3 Cardiology, Clinical Center University of Sarajevo, Sarajevo, BIH; 4 Cardiology, Sarajevo School of Science and Technology, Sarajevo, BIH; 5 Physical Medicine and Rehabilitation, University Clinical Center Tuzla, Tuzla, BIH

**Keywords:** acvr1 gene mutation, heterotopic ossifications, rehabilitation challenges, ultra-rare diseases, fibrodysplasia ossificans progressiva

## Abstract

Fibrodysplasia ossificans progressiva (FOP) is an exceptionally rare genetic disorder, representing humans’ most debilitating form of extraskeletal ossification. It is characterized by progressive postnatal heterotopic ossification of connective tissue and malformations of the big toes. In FOP, ectopic ossification usually begins in the upper paraspinal muscles and then spreads from axial to appendicular regions, cranial to caudal directions, and proximal to distal sites. The mean life expectancy for these patients is typically 40-50 years. Most patients need partial or complete assistance with walking by age 30, and common causes of death include thoracic insufficiency syndrome and pneumonia. We present the case of a patient with an advanced stage of FOP, highlighting its complex and progressive nature. The patient exhibits severe impairment of jaw mobility, swallowing difficulties, speech impediments, and hearing impairment. Additionally, severe kyphoscoliosis, heterotopic ossification of intercostal and paravertebral muscles, and ankylosis of the spine and all major joints of the upper and lower extremities, except the metacarpophalangeal and proximal interphalangeal joints, are evident. We discuss disease presentation, current management options, and rehabilitation challenges. To our knowledge, this is the first reported case of this rare disease from our country.

## Introduction

Fibrodysplasia ossificans progressiva (FOP) is an ultra-rare genetic disorder and the most disabling condition of extraskeletal ossification known in humans, characterized by postnatal progressive heterotopic ossification of the connective tissue and malformations of the big toes [1]. In 97% of cases, FOP arises from a de novo heterozygous mutation (c.617G>A; p.R206H) in the glycine and serine residue activation domain of activin A type I receptor (ACVR1)/activin-like kinase 2, a bone morphogenetic protein (BMP) type I receptor [2]. The mutation changes the ligand response profile of the ACVR1 receptor so that it becomes activated by the usually antagonistic activin A ligand, which leads to increased BMP signaling and abnormal bone formation [3]. There is also evidence of autosomal dominant inheritance with complete penetrance in some individuals. In these cases, FOP can be inherited from either parent, or there is a 50% chance that the child of an affected person will have the condition [4]. Recent studies indicate that the estimated prevalence of FOP may be higher than the previously referenced figure of 0.5 per million [5].

Individuals with this disorder are usually phenotypically indistinguishable from non-affected individuals at birth, except for the characteristic malformations of the great toes [1]. The spatial and temporal progression of ectopic ossification in FOP almost universally begins in the upper paraspinal muscles. It spreads later from axial to appendicular, cranial to caudal, and proximal to distal sites [6]. The mean life expectancy of these patients is limited to 40-50 years of age, with cardiorespiratory complications as the primary cause of death [7].

FOP is diagnosed clinically, with confirmation through genetic testing when possible. After discovering the genetic basis of the disorder, research into potential treatments for this severe condition has accelerated. In recent years, there has been no specific treatment for the disease, and therapy has been solely supportive. After its approval in Canada in 2022, palovarotene (marketed as Sohonos by Ipsen, based in Paris, France) was granted approval by the US FDA as a first treatment for FOP on August 16, 2023 [8]. Research on specific drug targets for FOP is still ongoing and promises a brighter future for patients. Rehabilitation should be included as a part of FOP patients’ medical care and must be carefully tailored, emphasizing enhanced daily activities.

We present the case of a patient in an advanced stage of FOP, with a short reference to rehabilitation challenges. To our knowledge, this is the first reported case of this rare disease from Bosnia and Herzegovina. The short version of this case report was presented as the poster for the 24th European Congress of Physical and Rehabilitation Medicine.

## Case presentation

A 35-year-old male patient who has been under our institution’s care for the past 15 years illustrates a complex and progressive disease course. He was born as the second child of a second uncomplicated pregnancy carried to term. The onset of symptoms occurred during early childhood as a painful swelling localized to the left shoulder at age two. He was diagnosed with FOP a year later. Over the past nine years, his mobility progressively declined, rendering him immobile for the last five years.

**Figure 1:**
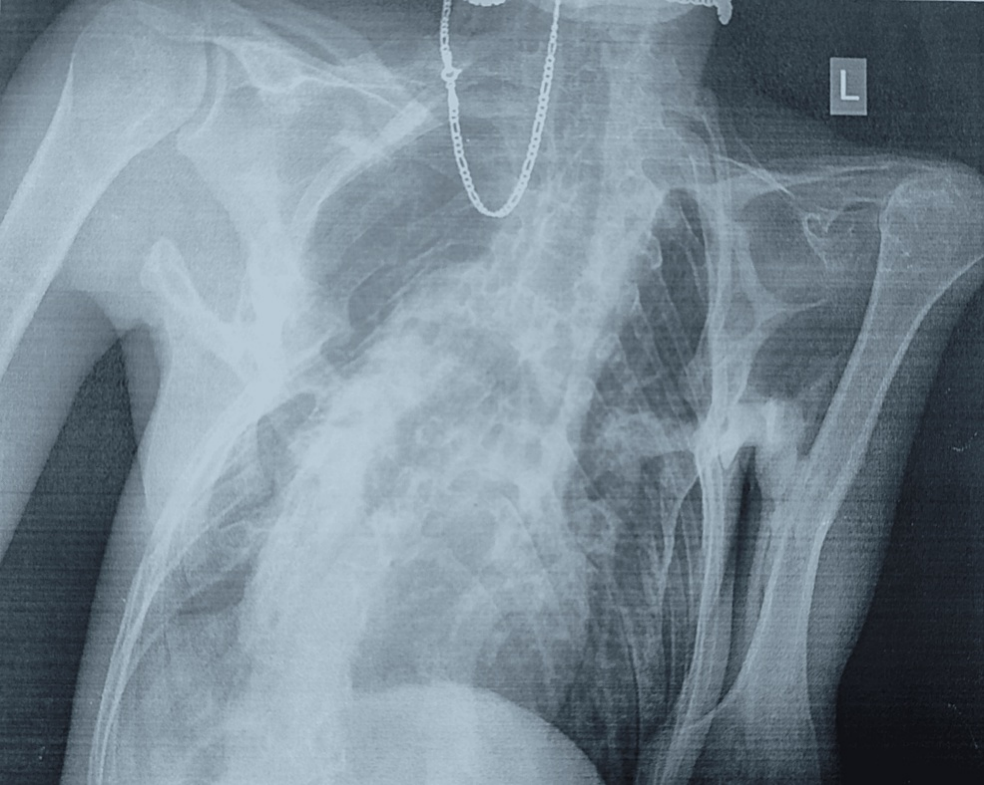
Chest X-ray in the supine position, with analysis significantly limited due to the patient’s forced posture. Fibrotic alteration in the lung parenchyma is observed, with decreased volume bilaterally, which is more pronounced on the right side. Additionally, reduced transparency is noted in the right hemithorax due to a summation effect caused by the patient’s rotation. The depicted segment of the thoracic spine exhibits dextroconvex scoliosis. Coarse shadows of ossifications corresponding to ossified ligaments are seen paravertebrally and in the medial aspect of the thoracic spine. Similar changes are observed bilaterally paravertebrally in the depicted cervical spine section. Ankylosis of the apophyseal joints is visible. In the soft tissues of the right thoracic wall, polymorphic ossifications leading to deformation of the chest wall are observed, with these ossifications merging with the arches of ribs II-VI. Ossifications are also seen in the left thoracic wall’s soft tissues at the axillary projection level, extending toward the left upper arm’s soft tissues and reaching the humeral diaphysis’s cortex.

Upon resuming balneophysical treatment after a two-year hiatus due to the pandemic, he remained immobile, requiring the aid of a backrest to achieve a semi-sitting position. Ankylosis of the temporomandibular joint and heterotopic ossifications within the masticatory muscles resulted in restricted mandibular movement and narrowing of the rima oris. A dental extraction procedure at age 19 exacerbated jaw mobility, further impairing swallowing and speech, necessitating a diet primarily composed of soft or pureed foods. Additionally, the patient experiences hearing impairment, a common feature of FOP. Severe kyphoscoliosis and heterotopic ossification of intercostal and paravertebral muscles contributed to the development of thoracic insufficiency syndrome (Figure 1).

**Figure 2:**
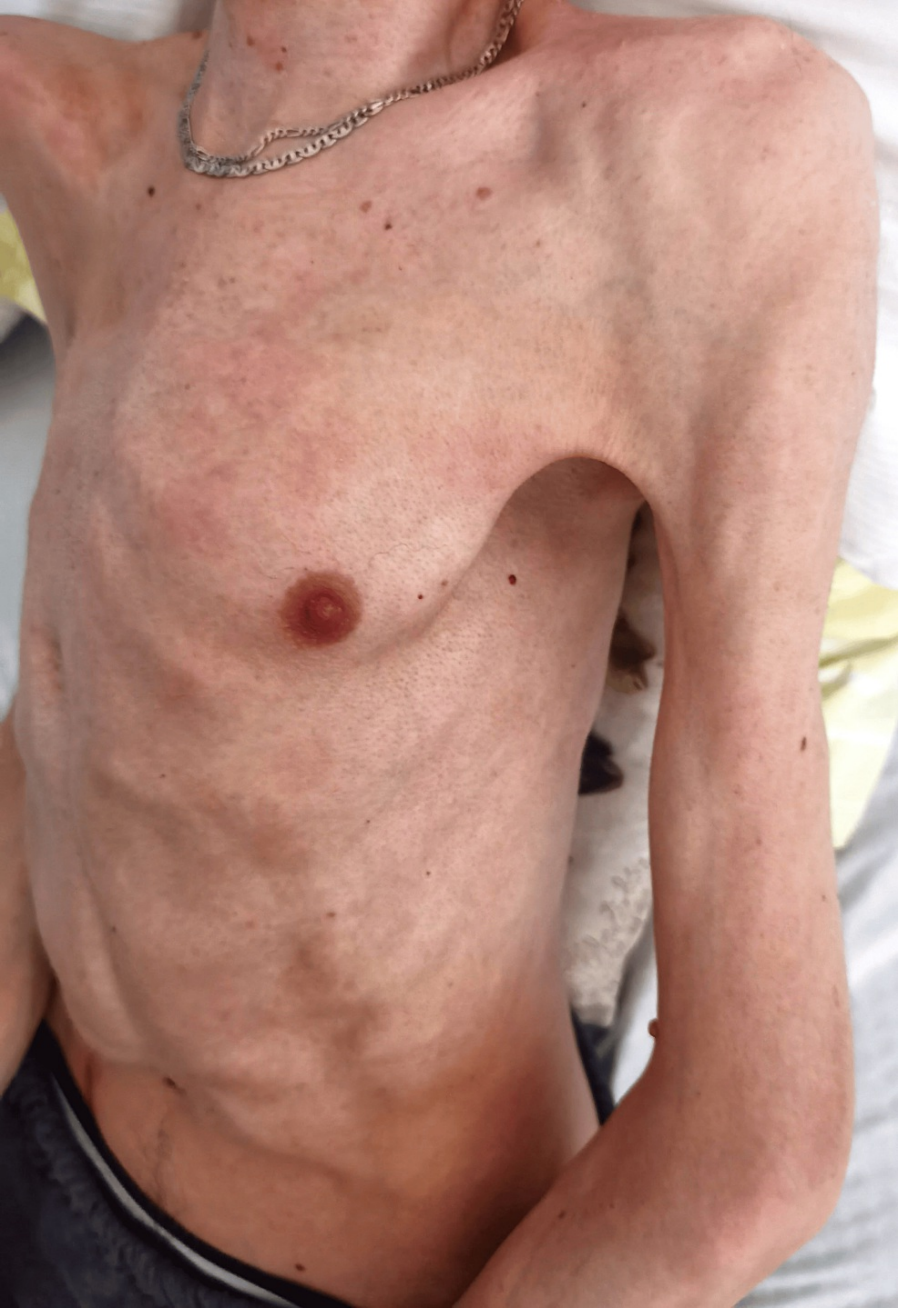
Ossifications within intercostal muscles; chest expansion is limited.

**Figure 3:**
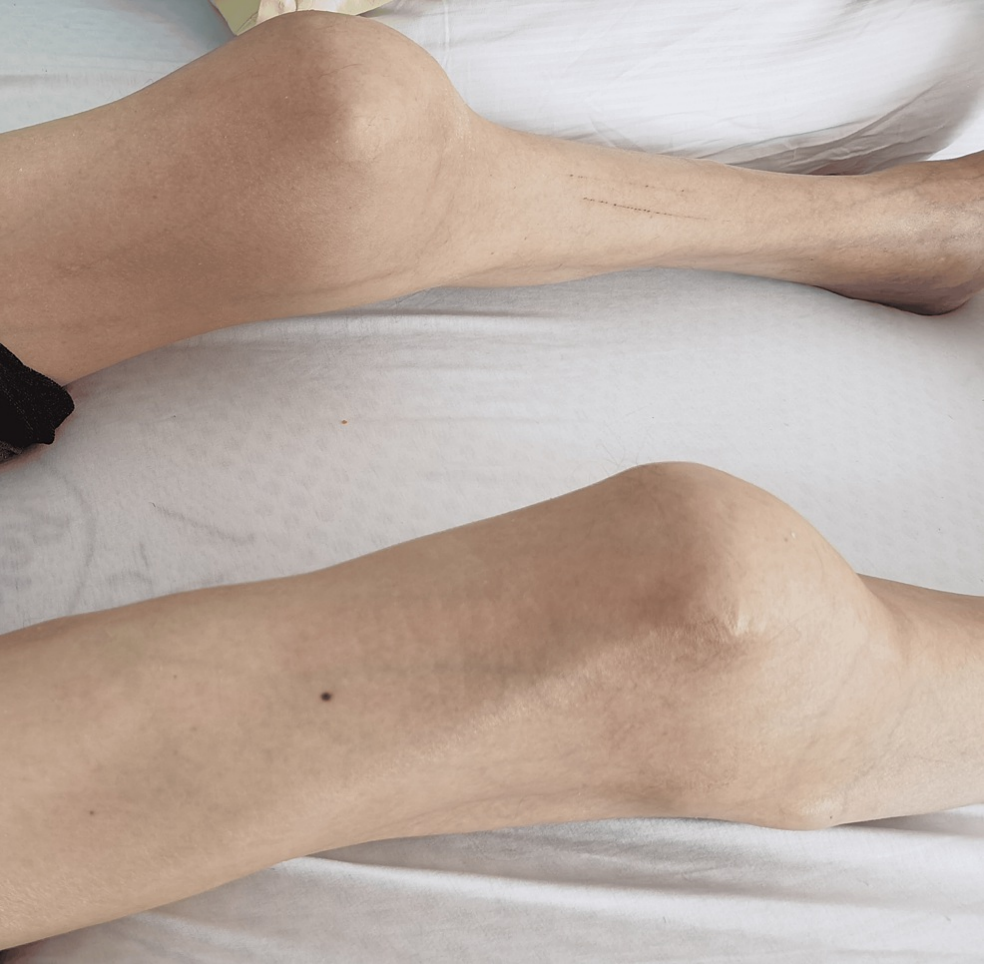
Ankylosis of knee joints

**Figure 4:**
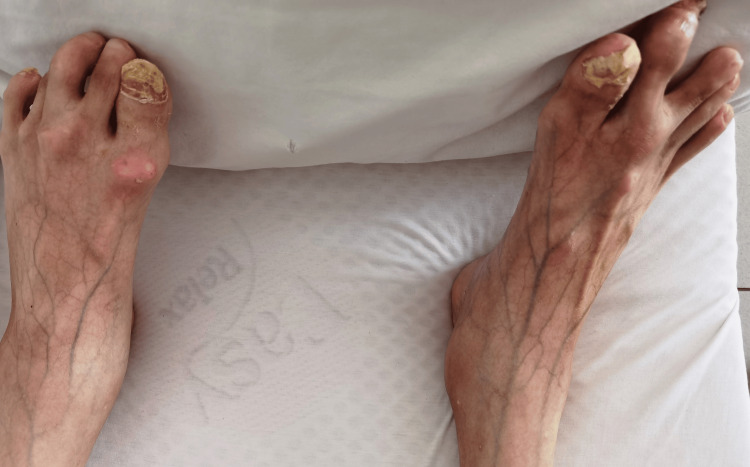
Shortened big toes, a hallmark of FOP FOP, fibrodysplasia ossificans progressiva

Chest expansion was limited (Figure 2), and he was dependent on diaphragmatic breathing. Among the upper and lower extremities, all joints were in ankylosis (Figure 3), except for the metacarpophalangeal (MCP) and proximal interphalangeal (PIP) joints of both hands, which retained some mobility, facilitating basic tasks such as grasping a mobile phone and accessing online sources. Extensive heterotopic ossifications were evident within the musculature of the upper arms, forearms, and posterior thigh muscles. Shortened big toes accompanied by multiple ossifications represented a characteristic hallmark of FOP (Figure 4).

The patient’s disease has reached an advanced stage, which makes rehabilitation challenging. Additionally, two years of pandemic-related limitations have further worsened the situation. Fortunately, the patient did not contract the virus, which could have posed a significant risk to his pulmonary function. Given that the patient has been under our care for an extended period, we provided education to the mother, who serves as the primary caregiver, regarding the progression of the disease, associated symptoms, and appropriate care measures. Upon admission, a thorough skin examination was conducted to check for pressure ulcers, and none were found. The patient was provided with an anti-decubitus mattress during previous visits. During the 10-day rehabilitation program at our center, he received specially prepared pureed food. He has used a Hubbard bath with crane assistance for the past five years. However, due to disease progression, this method could not be performed safely and effectively. Therefore, we opted for four-cell baths. The patient received mild-temperature four-cell thermo-mineral baths for 10 consecutive days for their upper and lower extremities. Additionally, gentle, active supported exercises were performed for the MCP and PIP joints of the upper extremities. Diaphragmatic breathing exercises were employed to enhance lung capacity, given that the diaphragm function remains intact. Despite facing significant challenges due to the progressive nature of the disease and limited rehabilitation options, the patient expressed satisfaction and maintained a positive mood throughout the process. Staying at the rehabilitation center and exposure to a different environment proved beneficial for the patient’s mental well-being, considering he spends most of his time at home.

## Discussion

The earliest description of the disease is credited to Guy Patin in 1692, when he wrote about a woman “who turned to wood” [9]. The London surgeon John Freke made the first clear description in 1736. He wrote, “There came a boy of healthy look and 14 years of age to ask of us at the hospital what should be done to cure him of many large swellings on his back, which began about three years since, and have continued to grow as large on many parts as a penny-loaf, particularly on the left side. They arise from all the vertebrae of the neck and reach down to the os sacrum. They likewise arise from every rib of his body, and joining together in all parts of his back, as the ramifications of coral do, they make, as it were, a fixed bony pair of bodice” [10]. Characteristic digital anomalies are present in all patients with FOP. Most commonly, the big toes are shortened with or without valgus deviation, and radiographs reveal deformity of the first metatarsals and a single phalanx in each big toe [11]. Hand changes are less frequent, including short first metacarpals and brachymesophalangy of the fifth finger with clinodactyly [12]. In our case, the mother reported that the patient had had shortened big toes since birth. Cervical spine abnormalities include large posterior elements, tall, narrow vertebral bodies, and fusion of the facet joints between C2 and C7 [13]. These congenital anomalies offer key diagnostic clues that are often overlooked or missed. Diagnostic errors frequently occur with FOP and are usually linked to inappropriate and potentially harmful diagnostic and therapeutic interventions. In a study involving 269 patients, initial incorrect diagnoses were given to 87% of them, with cancer being the most common misdiagnosis (32%). Among these patients, unnecessary invasive procedures (biopsies) were performed on 67%, and 68% received inappropriate therapies [14].

Ectopic ossification, another disease hallmark, occurs lifelong, with records of its initial appearance at the mean age of three or five years [12]. Notably, our patient experienced the first episode at age two. He has a hearing impairment, another common feature of FOP. Typically, it manifests as conductive hearing loss, possibly stemming from ossification in the middle ear. However, some individuals experience sensorineural hearing impairment, affecting the inner ear, cochlea, or auditory nerve [15]. The disease typically spares specific skeletal muscles, such as the diaphragm, tongue, and extraocular muscles. Cardiac and smooth muscles remain unaffected [16]. Although the diaphragm never ossifies in FOP, ankylosis of the costovertebral joints with chest wall fixation is present relatively early in the course of the disease. Severe upper thoracic kyphoscoliosis is a recognized cause of chronic respiratory failure and pneumonia [11]. In a study that included 60 patients, the most common causes of death in patients with FOP were cardiorespiratory failure from thoracic insufficiency syndrome (54%), pneumonia (15%), and complications of falls (11%) [7]. Proactive steps to optimize pulmonary function, reduce respiratory issues, and prevent influenza and pneumonia can significantly reduce the morbidity and mortality associated with thoracic insufficiency syndrome in patients with FOP [1].

Flare-ups of disease can manifest spontaneously or be triggered by trauma, including intramuscular injections such as vaccines, local anesthesia, particularly truncal blocks near the temporomandibular joint, muscle biopsies, and inadvertent venipuncture [12]. The results of a worldwide, prospective, cross-sectional survey of flare-ups in FOP patients have shown that approximately 88% of patients reported that at least some of their flare-ups occurred due to injury, viral infection, or overuse. Intramuscular immunization was the cause of flare-up in 25.1%, which led to bone formation in 84.3% of these patients. As many as 61% of all flare-ups occurred spontaneously [17]. As part of dental care, caries prevention is essential, and it is advisable to avoid intramuscular injections of local anesthetics, mandibular blocks, and excessive jaw stretching whenever possible [1]. Our patient had a flare-up of the disease, leading to limited jaw mobility after a tooth extraction.

Management of disease includes avoiding procedures that predispose flare-ups. Short-term high-dose corticosteroids may help reduce intense inflammation and tissue edema when used in the early stages of flare-ups [18]. Other medications, such as non-steroidal anti-inflammatory drugs, cyclooxygenase 2 inhibitors, mast cell inhibitors, and muscle relaxants, may be used to treat subsequent flare-ups [18,19]. Palovarotene, a retinoic acid receptor γ (RARγ) agonist that inhibits BMP signaling by binding to RARγ, was approved by the US FDA on August 26, 2023, as a treatment option for FOP. The drug is now approved for use in females aged eight years and older and in males aged 10 years and older [8]. Strategies such as gene therapy, small molecules, stem cell-based approaches, immunotherapy, and nanoparticle delivery systems are actively being investigated to target the underlying mechanisms of FOP and inhibit abnormal bone formation [19].

Rehabilitation should be focused on enhancing daily activities. Occupational therapy and vocational education consultations may be helpful [1]. Adapting the living space for someone with FOP is crucial, including wider corridors, minimal stairs, and convenient access to the bathroom and kitchen. Toilet seats should be modified, and showers with benches or stools are preferred over bathtubs. Providing appropriate aids based on the individual’s mobility level is essential. Ergonomically designed cane handles or custom-molded grips help reduce potential hand, elbow, and shoulder trauma. Rubber tips or shock-absorbing alternatives enhance stability, especially on slippery surfaces or ice. Specially prepared pureed foods and adjusted eating utensils are necessary for those with swallowing difficulties. Clothing should be loose-fitting and free of unnecessary buttons [20].

In kinesiotherapy, passive stretching should be avoided to prevent flare-ups. Instead, active or gently supported exercises are recommended. Warm water hydrotherapy is beneficial. Buoyancy in water allows individuals to perform active range-of-motion, cardiopulmonary, and resistance exercises in a safe, low-impact environment [20]. Sometimes, as in our case, assistance such as a specially designed crane is needed to provide safe hydrotherapy.

## Conclusions

Identifying an FOP gene was a significant step toward unraveling the enigma surrounding this rare disease. New therapies are in development, offering hope for a brighter future in combating this disabling condition. Given the rarity of the effects of FOP, raising awareness about the disease among medical professionals, patients, and their families is imperative. Early diagnosis and injury prevention are essential for effectively managing the condition and preventing flare-ups. Procedures predisposed to soft-tissue injuries, such as intramuscular injections, vaccinations, dental procedures, passive stretching, biopsies, removal of heterotopic bone, and all nonemergent surgical procedures, should be avoided. Rehabilitation should be an integral part of the overall medical care for FOP patients, incorporating occupational therapy, hydrotherapy, and hidrokinesitherapy.
